# Antibodies induced by antigen-containing liposomes as immunogens preferentially recognize their antigens present on lipid vesicles

**DOI:** 10.1038/s41598-026-42358-6

**Published:** 2026-03-11

**Authors:** Tetsuya Okuda, Michiyo Maruyama

**Affiliations:** Molecular Biosystems Research Institute, National Institute of Advanced Industrial Science and Technology (AIST), 1-8-31 Midorigaoka, Ikeda, 563- 8577 Osaka Japan

**Keywords:** antibody, liposome, glycolipid, flow cytometry, extracellular vesicle, breast cancer, Biochemistry, Biological techniques, Biotechnology, Cancer, Cell biology, Immunology

## Abstract

We established a flow cytometry method to detect interactions between antibodies against glycosphingolipids (GSLs) and liposomes composed of phospholipids, cholesterol, and GSLs. Using this system, we found that antibodies induced by antigen-containing liposomes as immunogens bound preferentially to the antigens present on liposomes. Anti-GSL antibodies obtained using GSL-containing liposomes as immunogens exhibited greater sensitivity in detecting GSLs present on liposomes compared with antibodies obtained using cells or glycoproteins as immunogens. Further analyses using the breast cancer–associated glycolipid Globo-H showed that antibodies capable of detecting extracellular vesicles secreted by MCF-7 breast cancer cells could be induced by immunizing mice with Globo-H–containing liposomes. Our results indicate that antibodies obtained using antigen-containing liposomes as immunogens are suitable for detecting antigens present on liposomes, and such antibodies can be applied to the detection of specific antigens present on extracellular vesicles.

**Supplementary Information:**

The online version contains supplementary material available at 10.1038/s41598-026-42358-6.

## Introduction

Liposomes are artificially generated lipid particles based on phospholipids that can encapsulate drugs and nutrients, and they are used as delivery vehicles to transport these substances into tissues and cells in vivo^1,2^. Although liposomes have recently attracted attention as carriers for nucleic acid vaccines, they have a long history as antigen carriers. Originally, liposomes were used to study the immunogenicity of lipid haptens^3–5^. Later, they were used as immunity-inducing carriers for lipid antigens such as glycosphingolipids (GSLs)^6–9^.

GSLs are components of cell membranes, and molecular species with a variety of glycan structures have been reported^10^. Because the glycan structures of GSLs present on the surface of mammalian cells change depending on the cellular differentiation stage and pathological conditions, they are often used as markers to identify cells. GSLs are also antigenic; thus, a number of monoclonal antibodies (mAbs) that recognize specific glycan structures of GSLs have been generated. GSL-specific antibodies can be efficiently induced by immunizing mice with GSLs using liposome carriers^10^. We developed several mAbs that specifically recognize glycan structures of GSLs and glycoproteins as epitopes by immunizing mice with liposomes containing natural or synthetic GSLs^11–16^. As these mAbs were able to bind GSLs expressed on the cell surface, we speculated that they could also bind GSLs on the surface of liposomes.

In this study, we developed a flow cytometry assay that enables the detection of liposome-antibody interactions and analyzed the liposome-binding specificity of several anti-GSL mAbs obtained using cells, synthetic glycoproteins, and GSL-containing liposomes as immunogens. Compared with mAbs obtained using other immunogens, mAbs produced using GSL-containing liposomes as immunogens showed greater sensitivity in detecting GSL antigens present on liposomes. In mice immunized with liposomes containing Globo-H, a glycolipid antigen associated with breast cancer cells, antibodies against Globo-H present on liposomes were readily induced in the serum. From these immunized mice, mAbs that detect extracellular vesicles (EVs) secreted by Globo-H–expressing MCF-7 breast cancer cells were obtained. The results of this study indicate that the use of liposomes as antigen carriers allows for the efficient induction of antibodies that specifically recognize antigens present on liposomes and EVs.

## Results

### Flow cytometry settings

**Fig. 1:**
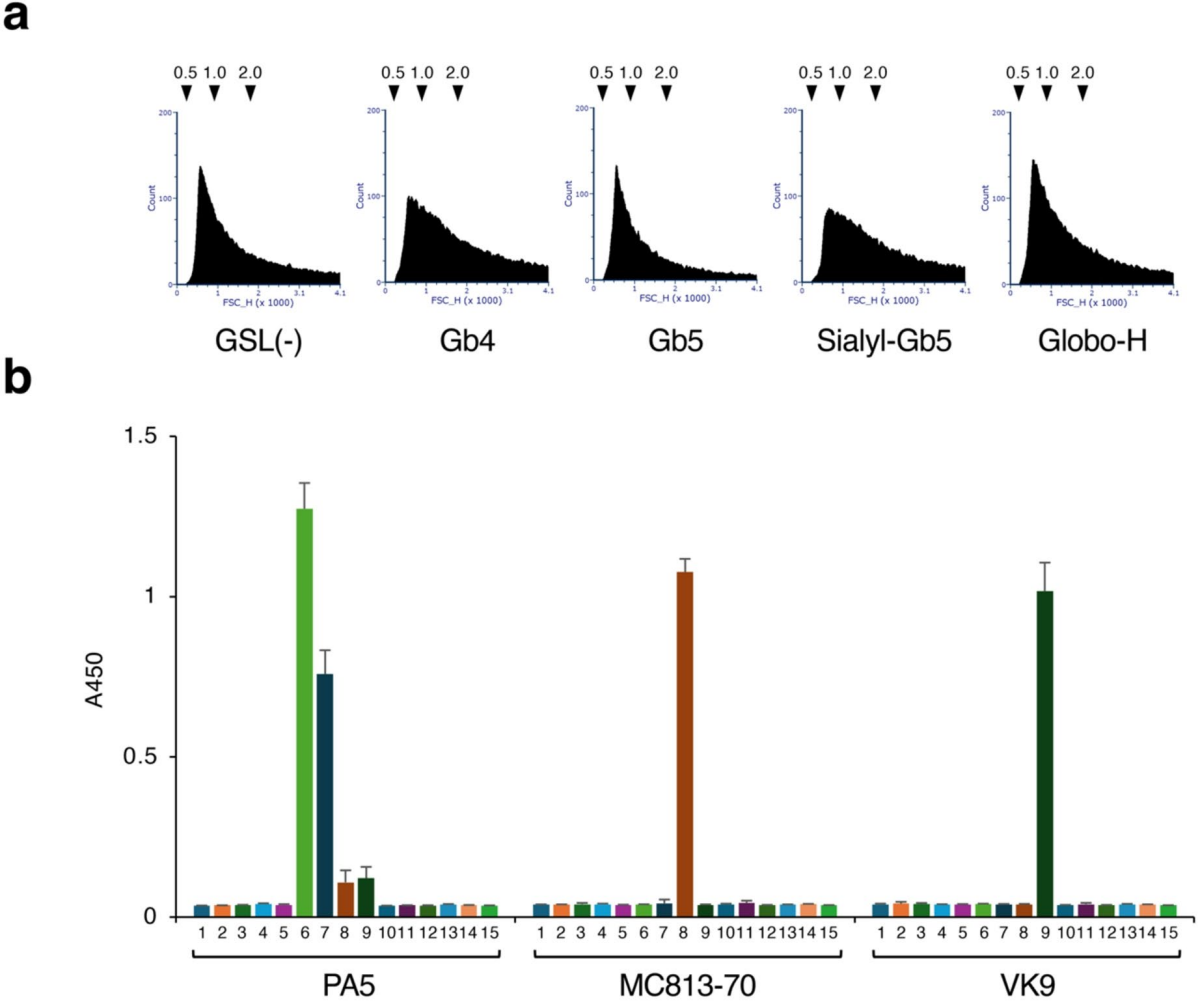
FCM analysis and ELISA of liposomes and antibodies used in this study. (**a**) The FSC signals of GSL-containing liposomes detected by FCM were visualized. The X- and Y-axes indicate the FSC signal intensity and the number of events, respectively. Arrowheads indicate the positions at which signals from 0.5-, 1.0-, and 2.0-µm standard beads were detected. Gb4, Gb5, Sialyl-Gb5, and Globo-H indicate liposomes containing the respective GSLs. GSL(−) indicates GSL-free liposomes. (**b**) Binding reactions of PA5, MC813-70, and VK9 mAbs to microtiter plate wells coated with various GSLs, as measured by ELISA. 1, Blank; 2, GlcCer; 3, GalCer; 4, LacCer; 5, Gb3; 6, Gb4; 7, Gb5; 8, Sialyl-Gb5; 9, Globo-H; 10, GM3; 11, GM2; 12, GM1; 13, GD1a; 14, GA2; 15, GA1. The chemical structures of these GSLs are shown in Supplementary Table S1. Error bars indicate the mean ± standard deviation. Data were obtained from two independent experiments.

Our previous dynamic light scattering analysis showed that liposomes prepared from dipalmitoylphosphatidylcholine (DPPC), cholesterol, and GSL formed a particle approximately 1000 nm in size in phosphate-buffered saline (PBS)^9^. Thus, in this study, we configured the flow cytometry (FCM) system to detect standard polystyrene beads with a diameter in the range of 500–2000 nm. To reduce background noise, the system was configured to not detect signals originating from beads with a diameter of 3000 nm or signals weaker than those originating from 500-nm beads (Fig. S1). Figure 1a shows the forward scattered light (FSC) signals of GSL liposomes detected using this FCM system. The GSL liposomes analyzed in this study were primarily composed of particles with a diameter between 500 and 1000 nm, although particles with a diameters of approximately 2000 nm were also detected in the analysis.

### Analysis of interactions between liposomes and antibodies

Three types of mAbs were used to analyze the interactions between these GSL liposomes and antibodies. PA5 is an IgM mAb that recognizes Gb4, which is known as globoside or the P blood group antigen. The PA5 mAb was isolated from mice immunized with Gb4-containing liposomes^9,11^. A specificity analysis using an enzyme-linked immunosorbent assay (ELISA) (Fig. 1b) revealed that PA5 also weakly reacts with Gb5, which is synthesized by adding a galactose group to Gb4 (Table S1). Gb5 is also known as the stem cell antigen SSEA-3. MC813-70 is an IgG3 mAb that recognizes Sialyl-Gb5, which is synthesized by adding a sialic acid group to Gb5. Sialyl-Gb5 is also known as the stem cell antigen SSEA-4. The MC813-70 mAb was isolated from mice immunized with human teratocarcinoma 2102Ep cells^17^. VK9 is an IgG3 mAb that recognizes Globo-H, which is synthesized by adding a fucose group to Gb5. The VK9 mAb was isolated from mice immunized with synthetic glycoproteins composed of Globo-H oligosaccharide and keyhole limpet hemocyanin^18^. ELISA results showed that these mAbs retained antigen-specific binding capability and detected the various antigens with comparable sensitivity (Fig. 1b).

**Fig. 2:**
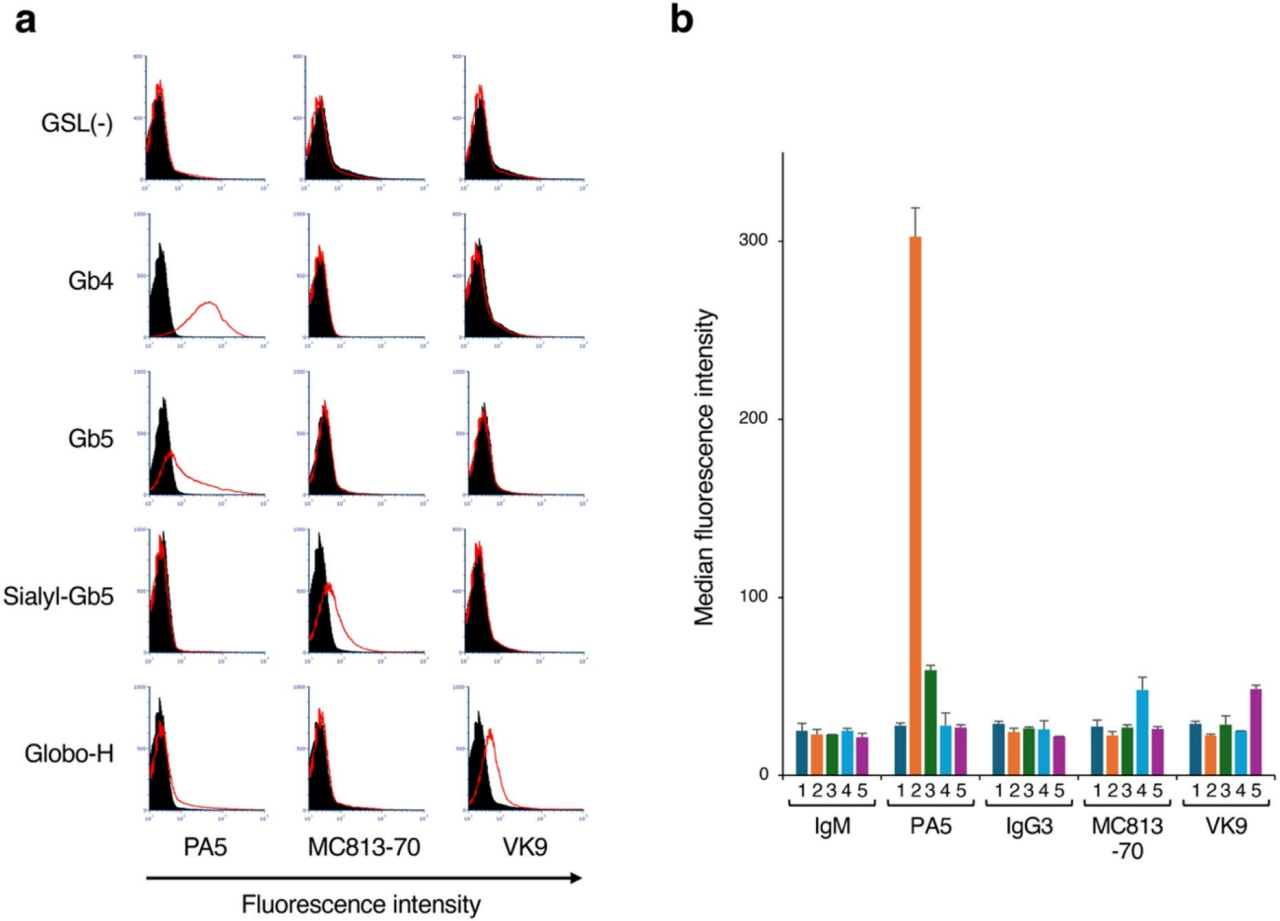
FCM analysis of the interactions between mAbs and liposomes. (**a**) FCM analysis images of the reactivity of PA5 (left panel), MC813-70 (center panel), and VK9 (right panel) mAbs to GSL-containing liposomes. Gb4, Gb5, Sialyl-Gb5, and Globo-H indicate liposomes containing the respective GSLs. GSL(−) indicate GSL-free liposomes. The X- and Y-axes indicate the fluorescence intensity and event number, respectively. The fluorescence intensity values of liposomes reacted with each antibody are shown by red lines. Controls for background monitoring were prepared using standard mouse IgM (for PA5) and IgG3 (for MC813-70 and VK9) and the corresponding fluorescently labeled secondary antibodies (dark shading). (**b**) MFI values measured by FCM from liposomes reacted with PA5, MC813-70, and VK9 mAbs. MFI values for liposomes reacted with standard IgM and IgG3 are shown as negative controls. 1, GSL-free liposomes; 2, Gb4-containing liposomes; 3, Gb5-containing liposomes; 4, Sialyl-Gb5–containing liposomes; 5, Globo-H–containing liposomes. Error bars indicate standard deviation.

The interactions between these antibodies and liposomes containing various types of GSLs were analyzed using the established FCM method (Fig. 2). Each antibody reacted only with the liposomes containing its corresponding antigen, demonstrating that in this assay, the antibodies maintain their specificity and selectively detect the target antigen present on liposomes (Fig. 2a). Next, to determine the sensitivity of each antibody for detecting the antigen present on liposomes, we compared median fluorescence intensity (MFI) values obtained from the FCM analysis (Fig. 2b). Only the PA5 mAb detected liposomes containing the target antigen with a significantly higher sensitivity than the other antibodies (Fig. 2b, PA5). Although MC813-70 and VK9 specifically reacted with liposomes containing the respective antigens, their MFI values were lower than that of PA5 (48.0 ± 7.1 and 48.5 ± 2.1, respectively). The MFI detected in the reaction with PA5 and Gb4-containing liposomes was significantly stronger (302.5 ± 16.3) compared with the other MFI values. These results indicate that when an antigen is administered to mice using liposomes as a carrier, the lymphocytes produce antibodies that preferentially recognize the antigen present on liposomes.

### Analysis of serum antibodies induced by Globo-H–containing liposomes

**Fig. 3:**
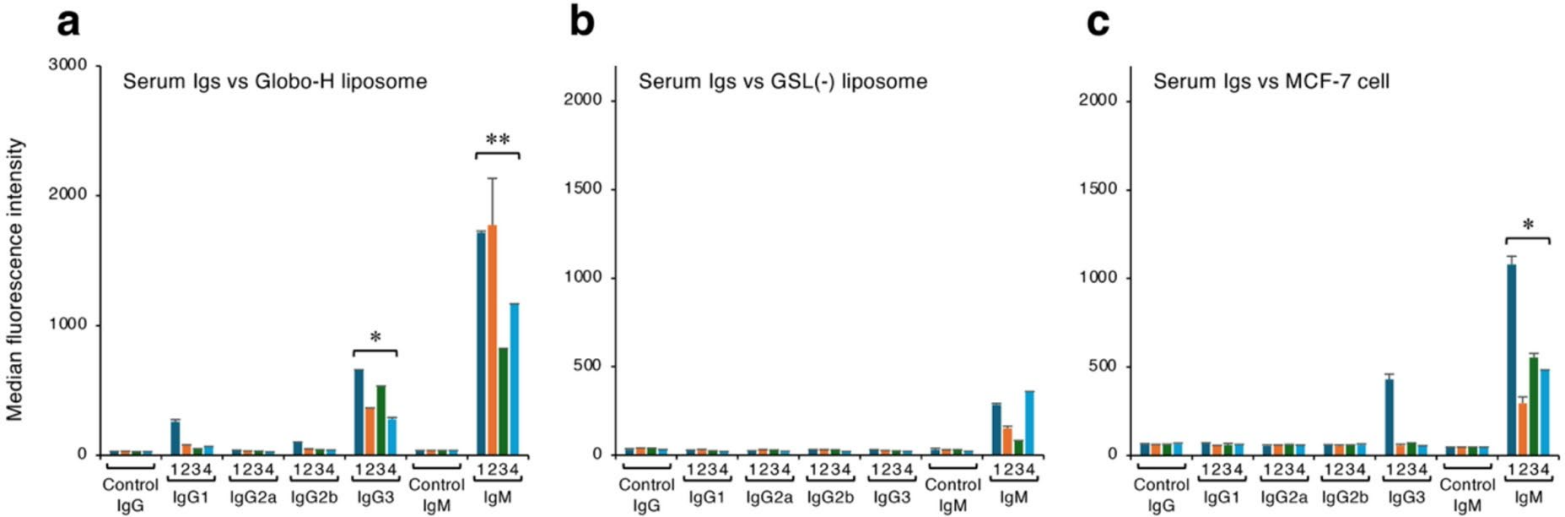
FCM analysis of serum antibodies from mice immunized with Globo-H–containing liposomes. MFI values for Globo-H–containing liposomes (**a**), GSL-free liposomes (**b**), and MCF-7 cells (**c**) reacted with serum antibodies from mice immunized with Globo-H–containing liposomes. Numerals 1, 2, 3, and 4 represent serum samples from different individual mice. Corresponding FCM images are shown in Supplementary Figures S2, S3, and S4. To detect the class/subclass of serum antibodies bound to liposomes, fluorescently labeled secondary antibodies capable of specifically detecting IgG1, IgG2a, IgG2b, IgG3, and IgM, respectively, were used. Control IgG and Control IgM represent controls for background monitoring; these were prepared using standard mouse total IgG and IgM instead of mouse serum and the corresponding fluorescently labeled secondary antibodies. Data were obtained from two independent experiments. *P < 0.05, **P < 0.01 control Ig group vs. serum antibody group (*n* = 4).

To verify that antigen administration using liposome carriers efficiently induces antibodies that preferentially bind to antigens present on liposomes, we analyzed serum antibodies of mice immunized with Globo-H–containing liposomes. The Globo-H used as a model in this study is specifically expressed in various cancer cells, including breast cancer cells^19–21^. We prepared serum antibodies from four mice repeatedly immunized with Globo-H–containing liposomes and then analyzed the interaction of these antibodies with Globo-H present on liposomes using FCM (Fig. 3). Analysis of serum obtained from the immunized mice revealed the presence of IgG1, IgG2b, IgG3, and IgM antibodies that bound to liposomes containing Globo-H (Figs. 3a and S2). Serum IgG1, IgG2b, and IgG3 in particular did not react with liposomes in the absence of Globo-H, indicating that these antibodies recognize Globo-H as an epitope present on liposomes (Figs. 3b and S3). Furthermore, analysis of MFI values showed that the IgG3 antibodies present in the serum detected Globo-H on liposomes with high sensitivity (mean MFI: 454.9 ± 169.4, *P* < 0.05 compared with the control IgG3 group). The MFI for the reaction between the IgMs and Globo-H–containing liposomes was even higher (mean MFI: 1369.4 ± 456.9, *P* < 0.01 compared with the control IgM group), but the IgMs also bound to liposomes without Globo-H (mean MFI: 218.3 ± 124.0). This result indicates that polyclonal IgMs consist of antibodies that bind non-specifically to liposomes and antibodies that recognize Globo-H present on liposomes.

**Fig. 4:**
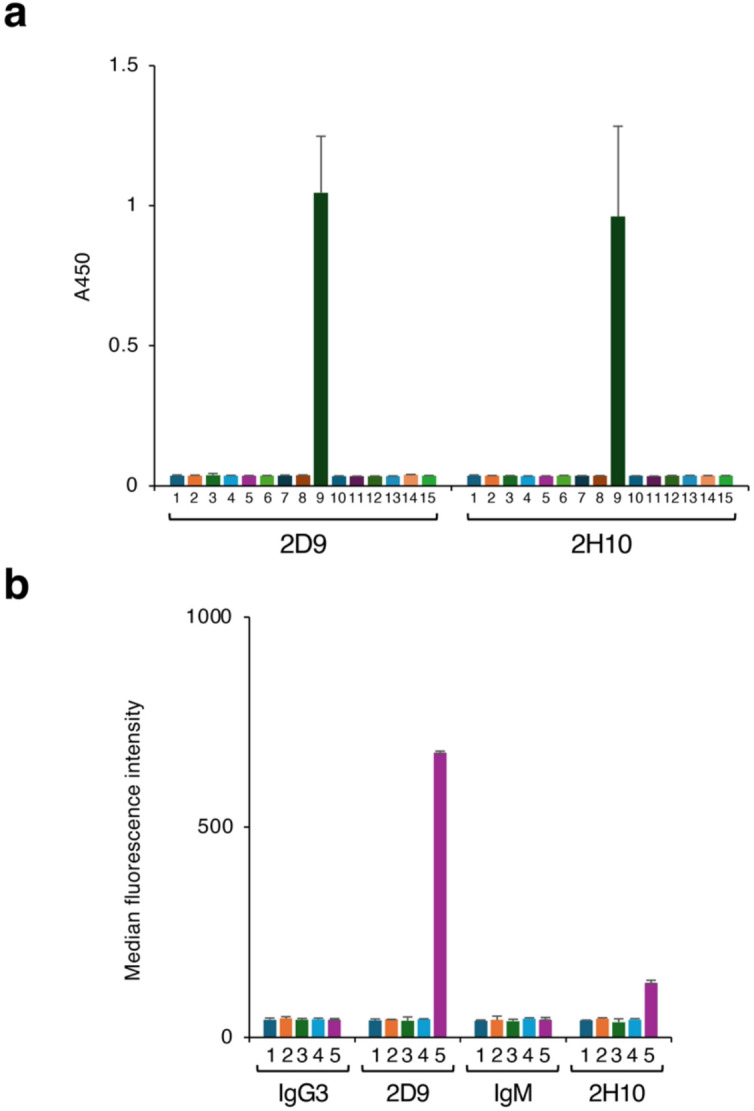
Specificity analysis of anti–Globo-H 2D9 and 2H10 mAbs. (**a**) The binding reactions of the 2D9 and 2H10 mAbs to microtiter plate wells coated with various GSLs were measured by ELISA. 1, Blank; 2, GlcCer; 3, GalCer; 4, LacCer; 5, Gb3; 6, Gb4; 7, Gb5; 8, Sialyl-Gb5; 9, Globo-H; 10, GM3; 11, GM2; 12, GM1; 13, GD1a; 14, GA2; 15, GA1. The chemical structures of the GSLs are shown in Supplementary Table S1. Error bars indicate the mean ± standard deviation. Data were obtained from two independent experiments. (**b**) MFI values in FCM analysis of liposomes reacted with 2D9 and 2H10 mAbs. 1, GSL-free liposomes; 2, Gb4-containing liposomes; 3, Gb5-containing liposomes; 4, Sialyl-Gb5–containing liposomes; 5, Globo-H–containing liposomes. MFI values for liposomes reacted with standard mouse IgG3 and IgM are shown as negative controls. Corresponding FCM images are shown in Supplementary Figure S5. Error bars indicate the mean ± standard deviation.

The reactivity of these serum antibodies to Globo-H expressed on the cell surface was analyzed using MCF-7 breast cancer cells that express Globo-H^19,20^. Although some individual variations were observed, both IgG3 and IgM present in the mouse serum clearly bound to these cells (Figs. 3c and S4). To analyze the specificity of these serum antibodies in greater detail, we generated two different mAbs*.* Spleen cells were collected from a mouse that produced serum antibodies that strongly reacted with liposomes and MCF-7 cells (No. 1 in Fig. 3), and hybridomas were generated using the collected splenocytes. From these hybridomas, two clones (2D9 and 2H10) were isolated that produce IgG3 or IgM class mAbs, respectively. These mAbs showed highly specific and sensitive reactivity toward Globo-H in ELISA (Fig. 4a). Next, we used FCM to analyze the reactivity of these antibodies to liposomes containing various GSLs (Fig. S5). Both antibodies bound only to liposomes containing Globo-H, consistent with the specificity observed in ELISA. Further analysis (Fig. 4b) showed that the 2D9 antibody detects Globo-H present on liposomes with very high sensitivity (MFI: 677.0 ± 4.2). The 2H10 mAb also clearly detected Globo-H present on liposomes (MFI: 129.5 ± 6.4), although the reactivity was relatively lower than that of the 2D9 mAb.

### Analysis of interactions between mAbs and EVs produced by MCF-7 cells

**Fig. 5:**
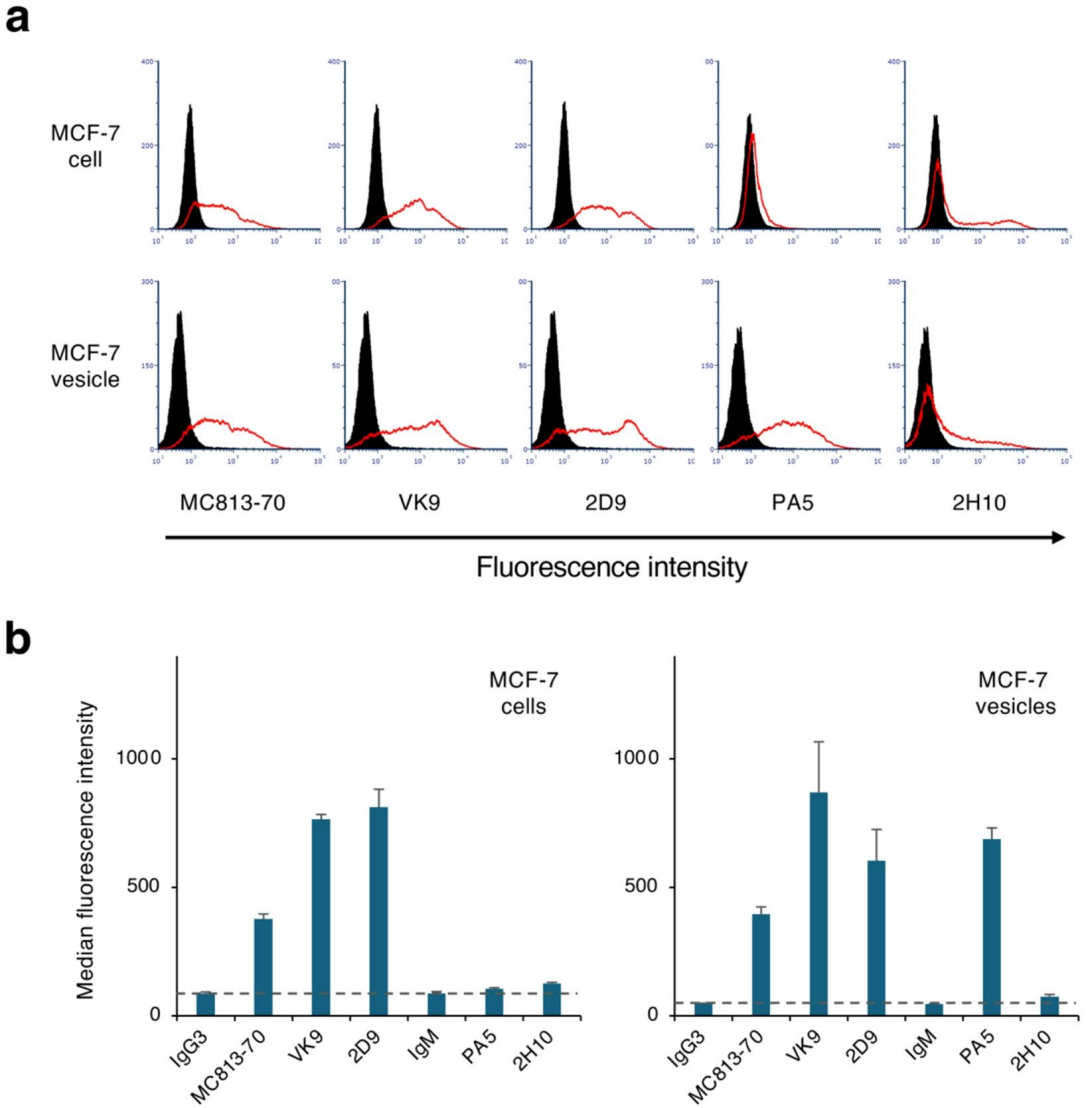
FCM analysis of EVs prepared from MCF-7 cells. (**a**) FCM analysis images of the reactivity of MC813-70, VK9, 2D9, PA5, and 2H10 mAbs to MCF-7 cells (upper panels) and MCF-7 cell–derived EVs (lower panels). The X- and Y-axes indicate the fluorescence intensity and event number, respectively. The fluorescence intensity values of samples reacted with each antibody are shown by red lines. Controls for background monitoring were prepared using standard mouse IgG3 (for MC813-70, VK9 and 2D9) or IgM (for PA5 and 2H10) and secondary antibodies (dark shading).(**b**) MFI values in FCM analysis of MCF-7 cells (left panel) and MCF-7 cell–derived EVs (right panel) reacted with each mAb. MFI values for each sample reacted with standard IgG3 and IgM are shown as negative controls. Error bars indicate standard deviation. Data were obtained from two independent experiments.

We hypothesized that antibodies capable of detecting antigens present on liposomes with high sensitivity might also preferentially bind to antigens present on lipid particles secreted by cells. To test this hypothesis, EVs secreted cells into the culture medium by MCF-7 were isolated using centrifugation, and interactions between the isolated EVs and the mAbs used in this study were evaluated using FCM. First, we analyzed the expression of GSL antigens on the cells (Fig. 5a, upper panels; Fig. 5b, left panel). FCM analysis showed that Globo-H, a well-characterized GSL expressed on MCF-7 cells, was detected by all three anti–Globo-H mAbs. Of these mAbs, VK9 and 2D9, which are IgG3 class, showed strong reactions to the cells, but the reaction of 2H10, an IgM class antibody, was weaker than that of the other mAbs. FCM analysis also showed that MCF-7 cells express Sialyl-Gb5, the epitope of the MC813-70 antibody. However, the epitopes of the PA5 antibody reacting with Gb4 and Gb5 were barely detectable, suggesting that Globo-H and Sialyl-Gb5 precursors are not expressed on the surface of these cells.

Next, we analyzed the reactivity of the mAbs with MCF-7 cell–derived EVs using FCM and detected binding of all mAbs to the EVs (Fig. 5a, lower panels; Fig. 5b, right panel). Even PA5, which showed low reactivity with MCF-7 cells (MFI: 104.5 ± 4.9), strongly reacted with the EVs (MFI: 686.8 ± 44.4). This strong reactivity of PA5 suggests that Gb4 and Gb5, which are contained within MCF-7 cells as Globo-H precursors, are secreted outside the cells as EVs. Further analysis of MFI values of the other samples showed that all of the mAbs examined except PA5 were able to detect MCF-7 cells and their EVs with similar reactivity. The VK9 and MC813-70 antibodies reacted weakly to their respective antigens present on liposomes but reacted strongly to the antigens when present on EVs. In contrast to this trend, 2D9 and PA5 reacted strongly with their respective antigens present on both liposomes and EVs. The 2H10 antibody clearly reacted with its antigen in ELISA, but its reactivity to antigens present on liposomes or cells and EVs was mild or weak, respectively. These results indicate that the binding of these mAbs to their antigens is significantly affected by whether the antigens are present on liposomes, cells, or EVs.

**Fig. 6:**
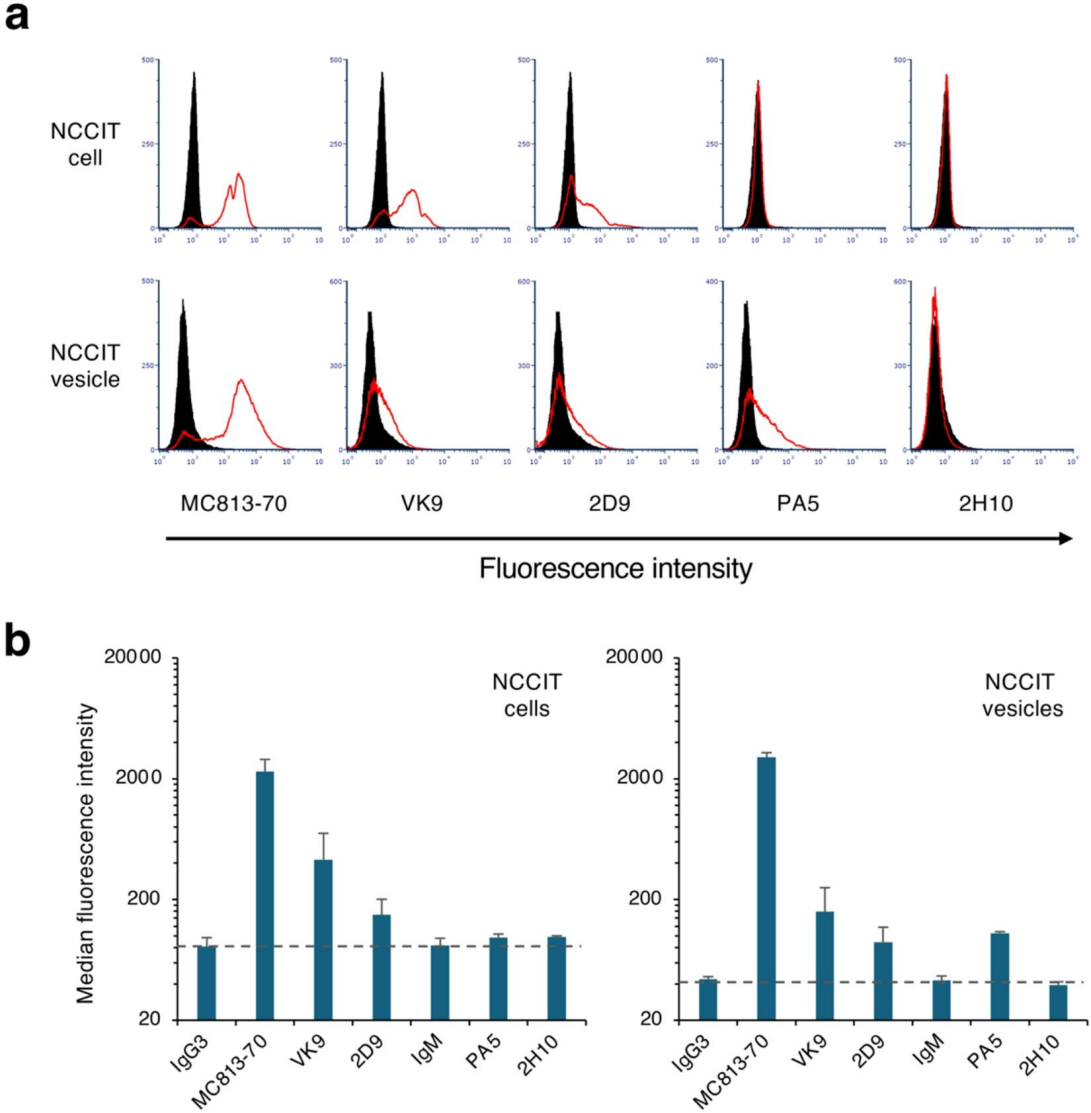
FCM analysis of EVs prepared from NCCIT cells. (**a**) FCM analysis images of the reactivity of MC813-70, VK9, 2D9, PA5, and 2H10 mAbs to NCCIT cells (upper panels) and NCCIT cell–derived EVs (lower panels). The X- and Y-axes indicate the fluorescence intensity and event number, respectively. The Y-axis is a logarithmic scale. The fluorescence intensity values of samples reacted with each antibody are shown by red lines. Controls for background monitoring were prepared using standard mouse IgG3 (for MC813-70, VK9, and 2D9) or IgM (for PA5 and 2H10) and secondary antibodies (dark shading). (**b**) MFI values in FCM analysis of NCCIT cells (left panel) and NCCIT cell–derived EVs (right panel) reacted with each mAb. MFI values for each sample reacted with standard IgG3 and IgM are shown as negative controls. Error bars indicate standard deviation. Data were obtained from two independent experiments.

We further investigated whether similar results could be observed in other cells (Fig. 6). For this analysis, we used human teratocarcinoma NCCIT cells, which are well known to express SSEA-4. MC813-70 antibody, which recognizes the SSEA-4 epitope, strongly reacted with the cell surface of NCCIT cells (MFI: 2998.5 ± 589.7) and with EVs produced by these cells (MFI: 2998.0 ± 287.7). VK9 and 2D9 antibodies also clearly reacted with the cell surface of NCCIT cells (MFI: 428.0 ± 277.9 and 149.3 ± 51.6, respectively) and with EVs produced by the cells (MFI: 158.8 ± 91.7 and 88.8 ± 29.1, respectively). Similar to its reactivity with MCF-7 cells, PA5 antibody did not react with the cell surface of NCCIT cells, but clearly reacted with EVs produced by the cells (MFI: 105.5 ± 2.6). 2H10 antibody did not react with NCCIT cells or EVs released from the cells, likely due to the lower expression level of Globo-H in NCCIT cells than in MCF-7 cells. This analysis revealed that SSEA-4, Globo-H, and Gb4 were expressed in NCCIT cells, and EVs produced by the cells could be detected using the MC813-70, VK9/2D9, and PA5 antibodies, respectively. These mAbs showed properties consistent with their reactivity observed using MCF-7 cells.

Based on the properties of the 2D9 and PA5 antibodies, we concluded that using antigen-containing liposomes as immunogens is an efficient method for obtaining antibodies capable of highly sensitive detection of antigens contained in EVs. Specifically, by screening antibodies that react strongly with target cells from antibodies induced by GSL-containing liposomes, it is possible to efficiently obtain antibodies that react strongly with EVs produced by these cells.

## Discussion

Here, we showed that antigen-containing liposomes efficiently induce antibodies that react with antigens present on certain types of lipid vesicles, such as artificially prepared liposomes and EVs. In particular, we demonstrated that GSL-containing liposomes can be used as immunogens to generate antibodies that specifically detect GSL antigens present on EVs. By combining this immunization method with a method for screening induced antibodies that strongly react with cells expressing target GSL, it is possible to efficiently obtain antibodies that recognize EVs secreted from target cells. As EVs function as a medium for intercellular communication and regulate a variety of biological responses^22,23^, antibodies obtained using this method will be useful tools for studying the physiology of EVs and related diseases. Another advantage of GSL-containing liposomes is that they can induce antibodies against GSLs more efficiently than methods using synthetic glycoproteins or cells as immunogens. When protein carriers or cells are used as immunogens, antibodies to protein components are also induced, resulting in a lower proportion of the target GSL antibodies among the induced antibodies. By contrast, phospholipids and cholesterol used as liposome components induce a limited immune response in animals and therefore do not interfere with the induction and isolation of antibodies against the target GSLs contained in the liposomes.

Our established FCM method for detecting GSLs offers various advantages over conventional methods, such as ELISA, thin-layer chromatography, and surface plasmon resonance. For example, ELISA can lead to inaccurate assessment of antibody reactivity against GSLs due to signal saturation, whereas FCM analysis using fluorescent probes can accurately analyze the strength of antibody reactivity against GSLs in a sample. Conventional methods also require highly purified GSLs from samples prior to analysis, as the presence of other contaminating molecules reduces detection sensitivity of GSL components. By contrast, the FCM method allows the precipitate obtained by centrifugation to be used directly for analysis, enabling GSL analysis in a shorter and simpler procedure than existing methods. This property also makes them suitable for antibody detection of GSLs in biological membranes where other molecules such as phospholipids, proteins, nucleic acids, and steroids are present, potentially enabling applications in pathological diagnostics such as liquid biopsies.

Mice immunized with liposomes containing Globo-H as an antigen produced various classes of antibodies against liposomes in the serum, but only the IgG3 and IgM classes of these antibodies reacted with MCF-7 cells expressing Globo-H. This result indicates that the antibodies induced by Globo-H–containing liposomes include antibodies that react with Globo-H present on liposomes but not Globo-H expressed on cells. By contrast, the VK9 and MC813-70 mAbs obtained from mice immunized with synthetic glycoproteins or teratocarcinoma cells showed poor reactivity with artificially prepared liposomes containing their respective antigens but clearly reacted with MCF-7 and NCCIT cells expressing the antigens and with EVs secreted from these cells. These results indicate that the scaffold structure surrounding the antigen has a significant impact on the antigen-antibody reaction on lipid vesicles. In the case of antibodies that recognize carbohydrate antigens as epitopes, cell-derived carbohydrate molecules not present in liposomes, such as glycoproteins present in both the cell membrane and EVs, may affect the reactivity of these antibodies against their respective antigens present on EVs.

The PA5 mAb, which reacted weakly with MCF-7 cells, reacted strongly with EVs derived from MCF-7 cells. Similarly, PA5 did not react with NCCIT cells, but clearly reacted with EVs derived from the cells. As the antigens of PA5 antibody (Gb4 and Gb5) are precursors for Globo-H and SSEA-4, the major GSLs expressed on the surface of these cells, these GSLs are primarily intracellular^20^. Thus, the strong reaction of PA5 with EVs secreted from these cells may reflect the intracellular membrane structure of these cells. These observations indicate that analyzing the constituent molecules of target EVs and reflecting this in the composition of liposomes used as immunogens will enable more efficient production of antibodies that can detect target EVs. Alternatively, the FCM method established in this study can also be used to screen for antibodies that strongly react with target EVs from antibodies induced by antigen-containing liposomes. The latter method is more practical for immunization experiments in which large amounts of immunogen are administered to animals, as materials for that method are more readily available than those for the former method.

Further research based on this study will lead to the development of anti–lipid vesicle antibodies, which are expected to become useful tools in a variety of fields, such as quality control of liposomes used in pharmaceuticals and the use of EVs in disease diagnosis. Also, these antibodies will serve as a tool in studies aimed at deepening our understanding of the molecular mechanisms of antibody recognition of antigens on lipid membranes, potentially leading to important findings in infectious disease and cancer treatment research.

## Methods

### Materials

GSLs were obtained from Tokyo Chemical Industry (Tokyo, Japan) or prepared as described previously^24^. MCF-7 human breast carcinoma cells, NCCIT human teratocarcinoma cells, and Sp2/0-Ag14 myeloma cells were obtained from the JCRB Cell Bank (Osaka, Japan), the American Type Culture Collection (Manassas, VA, USA), and Riken Cell Bank (Tsukuba, Japan), respectively. VK9 mouse monoclonal IgG3 and MC813-70 mouse monoclonal IgG3 were obtained from Thermo Fisher Scientific (Waltham, MA, USA) and BioLegend (San Diego, CA, USA), respectively. Alexa Fluor 488–conjugated anti-mouse, IgG1, IgG2a, IgG2b, IgG3, and IgM were obtained from Thermo Fisher Scientific. Horseradish peroxidase (HRP)-labeled goat, anti-mouse IgM, and anti-mouse IgG were obtained from Sigma-Aldrich (St. Louis, MO, USA). Mouse immunoglobulin standards were obtained from Wako (Osaka, Japan) or BML (Tokyo, Japan).

### Liposome preparation

Liposomes were prepared according to the Bangham method^25,26^. For FCM analyses, 2 µg of GSLs was mixed with 0.02 µmol of DPPC and 0.02 µmol of cholesterol in chloroform/methanol (2:1, *v/v*). For immunization, 50 µg of GSLs was mixed with 10 µg of lipid A, 0.5 µmol of DPPC, and 0.5 µmol of cholesterol in chloroform/methanol (2:1, *v/v*). The mixture was dried and then dissolved in 200 µl of PBS for FCM analysis or 500 µl for immunization and vortexed at 50 °C for 1 min to prepare liposomes.

### FCM settings and analysis of samples

For analysis of liposomes and EVs, the Gain/PMT voltage values of FSC, side scattered light (SSC), and fluorescence detector channel 1 (FL1) of the FCM (RF-500 flow cytometer, Sysmex, Tokyo, Japan) were set to 1000, 880, and 450, respectively, and the threshold for FSC only was set to 100. A gate was then set to detect only events within this range. For analysis of MCF-7 cells, the Gain/PMT voltage values of FSC, SSC, and FL1 were set to 140, 360, and 320, respectively, and then, using FSC and SSC as a guide, a gate was set to detect only the cell population. To detect GSL antigens on samples of liposomes, cells, and EVs, suspensions in PBS were incubated on ice with 2.5 µg of mAb or 10 µl of serum, followed by sequential labeling with class-specific Alexa Fluor 488–conjugated anti-mouse immunoglobulin (Thermo Fisher Scientific). Cells were pelleted by centrifugation at 200*g* f for 1 min and washed via buffer exchange. Liposome and EV samples were pelleted by centrifugation at 10,000*g* for 3 min and washed via buffer exchange. The pelleted samples were stirred into fresh PBS by tapping and analyzed by FCM. To calculate the MFI, 20,000 events for cells and 100,000 events for liposomes and EVs were analyzed. Generation of FCM images and calculation of MFI were performed using FCS express 7 flow cytometry software (De novo Software, Pasadena, CA, USA). All samples were analyzed in duplicate in each experiment.

### Maintenance of cells and preparation of EVs

MCF-7 cells were maintained on culture dishes (100 mm diameter) in E-MEM medium with non-essential amino acids (Wako) and containing 10% fetal bovine serum, 1 mM pyruvic acid, and 10 µg/ml insulin at 37 °C in a humidified atmosphere containing 5% CO_2_. NCCIT cells were maintained in RPMI-1640 medium (Wako) containing 10% fetal bovine serum, 100 units/ml penicillin, and 100 μg/ml streptomycin, with other conditions being the same as those for MCF-7 cells. To prepare EVs, 5 × 10^6^ cells were seeded onto the dish and maintained in 15 ml of culture medium for 2 days, at which time the medium was collected. The collected medium was first centrifuged at 200*g* for 4 min to remove cells and cell debris. The supernatant was then centrifuged and concentrated using a Spin-X UF20 concentrator filtration system (Corning Life Sciences, Tewksbury, MA, USA) at 3260*g* for 30 min. The resulting concentrate was further centrifuged at 10,000*g* for 3 min to prepare a precipitate. The precipitate was washed with PBS and then stirred in fresh PBS and used as the EV sample. For FCM analysis, prepared EVs or 1 × 10^6^ harvested cells were suspended in 200 µl of cold PBS and used as samples.

### Immunization and preparation of serum

Female C3H/HeN mice (CLEA Japan, Tokyo) were immunized with Globo-H–containing liposomes as described previously^8,12,16^. Mice were initially immunized subcutaneously and then intraperitoneally at 2 weeks after the first immunization. After two more immunizations, samples of blood and spleen were collected 3 days after the final immunization. To collect the samples, mice were placed in an anesthesia bottle saturated with 0.5 ml of isoflurane inhalation solution (Zoetis, Tokyo, Japan) and sacrificed by inhalation of vaporized isoflurane. The collected blood was incubated at 37 °C for 90 min, after which the clot was removed, the sample was centrifuged at 800*g* for 15 min, and the supernatant was collected as serum. All mice were housed in a controlled specific pathogen-free animal room at a temperature of 23 ± 2 °C and humidity of 55 ± 15% under a 12-h light/12-h dark cycle. The animals were given ad libitum access to food and water. All animal experiments were approved by the Committee for Experiments Involving Animals of the National Institute of Advanced Industrial Science and Technology (approval number: Animal2024-0119-B) and performed in accordance with the relevant guidelines and regulations. Findings were reported in accordance with ARRIVE guidelines^27^.

### Hybridoma generation and characterization of immunoglobulin isotypes

Splenocytes collected from a mouse immunized with Glob-H–containing liposomes were fused with Sp2/0-Ag14 myeloma cells as described previously^12,16^. The fused cells were then seeded into 96-well microtiter plates to grow as a single colony per well, and hybridomas were selected using a hypoxanthine-aminopterin-thymidine selection medium (RPMI-1640 containing 10% fetal calf serum, 0.1 mM sodium hypoxanthine, 0.4 µM aminopterin, 16 µM thymidine, 10 µg/ml gentamicin, and 5% Briclone [KAC Co., Ltd., Hyogo, Japan]). The cells were cultured at 37 °C in a humidified atmosphere containing 5% CO_2_. Culture supernatants were evaluated by ELISA, and clones showing positive reactivity against Globo-H were selected. The isotype of monoclonal antibody was determined using a mouse monoclonal antibody isotyping kit (Roche Diagnostics GmbH, Mannheim, Germany).

### ELISA

ELISAs were performed as described previously^24^. A total of 500 ng of GSLs dissolved in methanol was added to each well of a 96-well microtiter plate and incubated and allowed to dry for fixation onto the plate. Blocking buffer (1% bovine serum albumin in PBS) was then added to the antigen-coated wells and incubated for 15 min at room temperature, followed by the addition of 1 µg/ml mAbs or 50-fold diluted serum. After incubation for 2.5 h at room temperature, the wells were washed with 0.05% Tween 20 in PBS, and then HRP-labeled secondary antibodies were added. Binding of HRP-labeled secondary antibody to the primary antibody was detected using an HRP substrate (1-Step Ultra TMB-ELISA Substrate; Thermo Fisher Scientific), and the absorbance was measured at 450 nm. Samples were analyzed in duplicate in each experiment.

### Statistical analysis

After determination of variance using the F-test, statistical significance was determined using the two-tailed Welch’s *t*-test, with statistical significance defined as follows: *P < 0.05, **P < 0.01.

## Supplementary Information

Below is the link to the electronic supplementary material.

Supplementary Material 1

## Data Availability

The datasets generated during and/or analyzed during the current study are available from the corresponding author on reasonable request.
