# Supplementary material for: Antibodies induced by antigen-containing liposomes as immunogens preferentially recognize their antigens present on lipid vesicles
Source: Sci Rep. 2026 Mar 11;16:13161. doi: 10.1038/s41598-026-42358-6 (PMC13103445; doi:10.1038/s41598-026-42358-6)
Supplement: Supplementary file 1 — Supplementary Material 1 [file 41598_2026_42358_MOESM1_ESM.docx]

**Supplementary Information**

Table S1.

**Chemical structures of glycosphingolipids used in this study.**

Abbreviation Structure

GlcCer Glcβ1,1Cer

GalCer Galβ1,1Cer

LacCer Galβ1,4Glcβ1,1Cer

Gb3 Galα1,4Galβ1,4Glcβ1,1Cer

Gb4 GalNAcβ1,3Galα1,4Galβ1,4Glcβ1,1Cer

Gb5 Galβ1,3GalNAcβ1,3Galα1,4Galβ1,4Glcβ1,1Cer

Sialyl-Gb5 Siaα2,3Galβ1,3GalNAcβ1,3Galα1,4Galβ1,4Glcβ1,1Cer

Globo-H Fucα1,2Galβ1,3GalNAcβ1,3Galα1,4Galβ1,4Glcβ1,1Cer

GM3 Siaα2,3Galβ1,4Glcβ1,1Cer

GM2 GalNAcβ1,4(Siaα2,3)Galβ1,4Glcβ1,1Cer

GM1 Galβ1,3GalNAcβ1,4(Siaα2,3)Galβ1,4Glcβ1,1Cer

GD1a Siaα2,3Galβ1,3GalNAcβ1,4(Siaα2,3)Galβ1,4Glcβ1,1Cer

GA2 GalNAcβ1,4Galβ1,4Glcβ1,1Cer

GA1 Galβ1,3GalNAcβ1,4Galβ1,4Glcβ1,1Cer

Abbreviations: Glc, glucose; Cer, ceramide; Gal, galactose; GalNAc, *N*-acetylgalactosamine; Sia, sialic acid (Neu5Ac); Fuc, fucose.


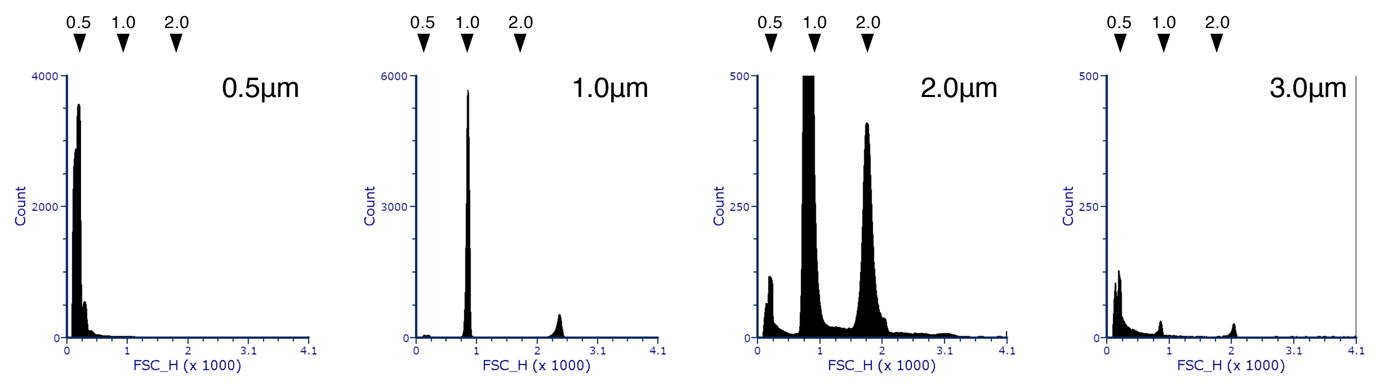


Fig. S1.

**FSC signals of standard polystyrene beads.**

The X- and Y-axes show the FSC signal intensity and number of events, respectively. Arrowheads indicate the positions of the main signal peaks for 0.5-, 1.0-, and 2.0-μm standard beads.

With the FSC settings used in this study, the main signals for 3.0-μm beads were outside the detection range. As several signal peaks were detected from the 2.0-μm beads, the peak that did not overlap with the peaks of the 0.5-μm and 1.0-μm beads was determined to be the main signal peak for the 2.0-μm beads.


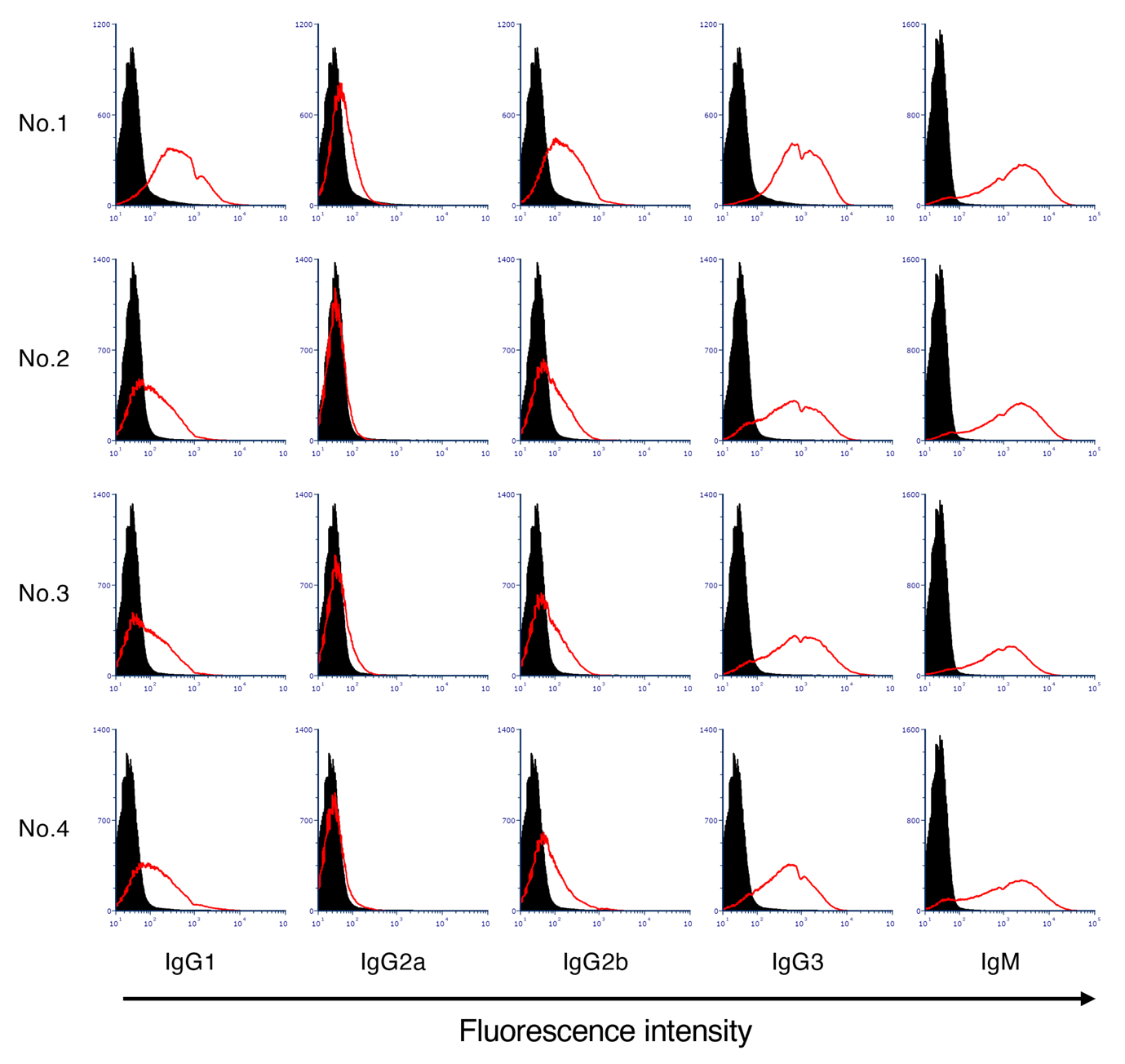


Fig. S2

**FCM analysis images of the reactivity to Globo-H–containing liposomes of serum antibodies from mice immunized with Globo-H–containing liposomes.**

Nos. 1, 2, 3, and 4 represent serum samples from different individual mice. The X- and Y-axes indicate the fluorescence intensity and event number, respectively. The fluorescence intensity values of liposomes reacted with each serum sample are indicated by red lines. Controls for background monitoring were prepared using standard total mouse IgG and IgM and the corresponding fluorescently labeled secondary antibodies (dark shading).


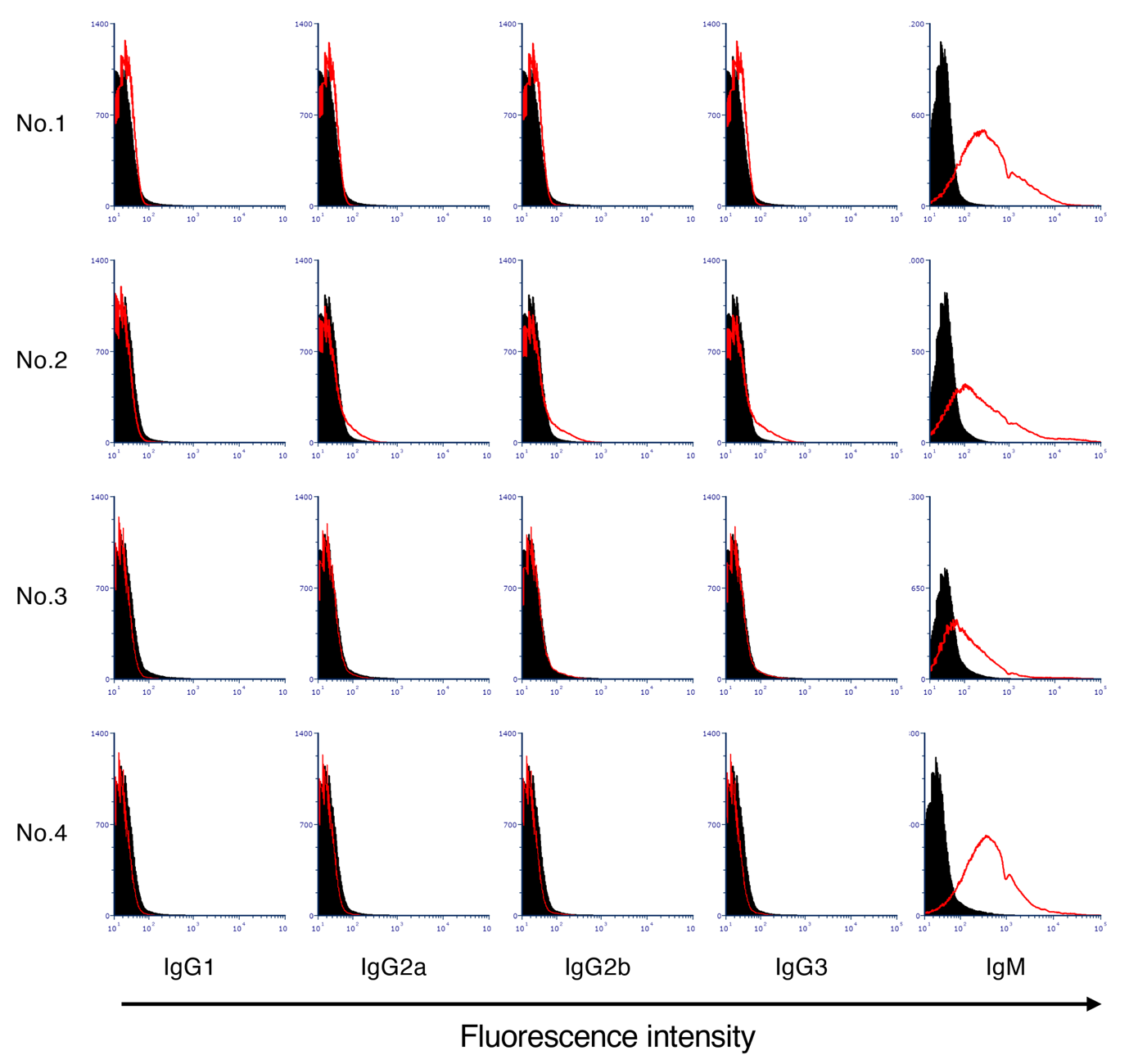


Fig. S3

**FCM analysis images of the reactivity to GSL-free liposomes of serum antibodies from mice immunized with Globo-H–containing liposomes.**

Nos. 1, 2, 3, and 4 represent serum samples from different individual mice. The X- and Y-axes indicate the fluorescence intensity and event number, respectively. The fluorescence intensity values of liposomes reacted with each serum sample are indicated by red lines. Controls for background monitoring were prepared using standard total mouse IgG and IgM and the corresponding fluorescently labeled secondary antibodies (dark shading).


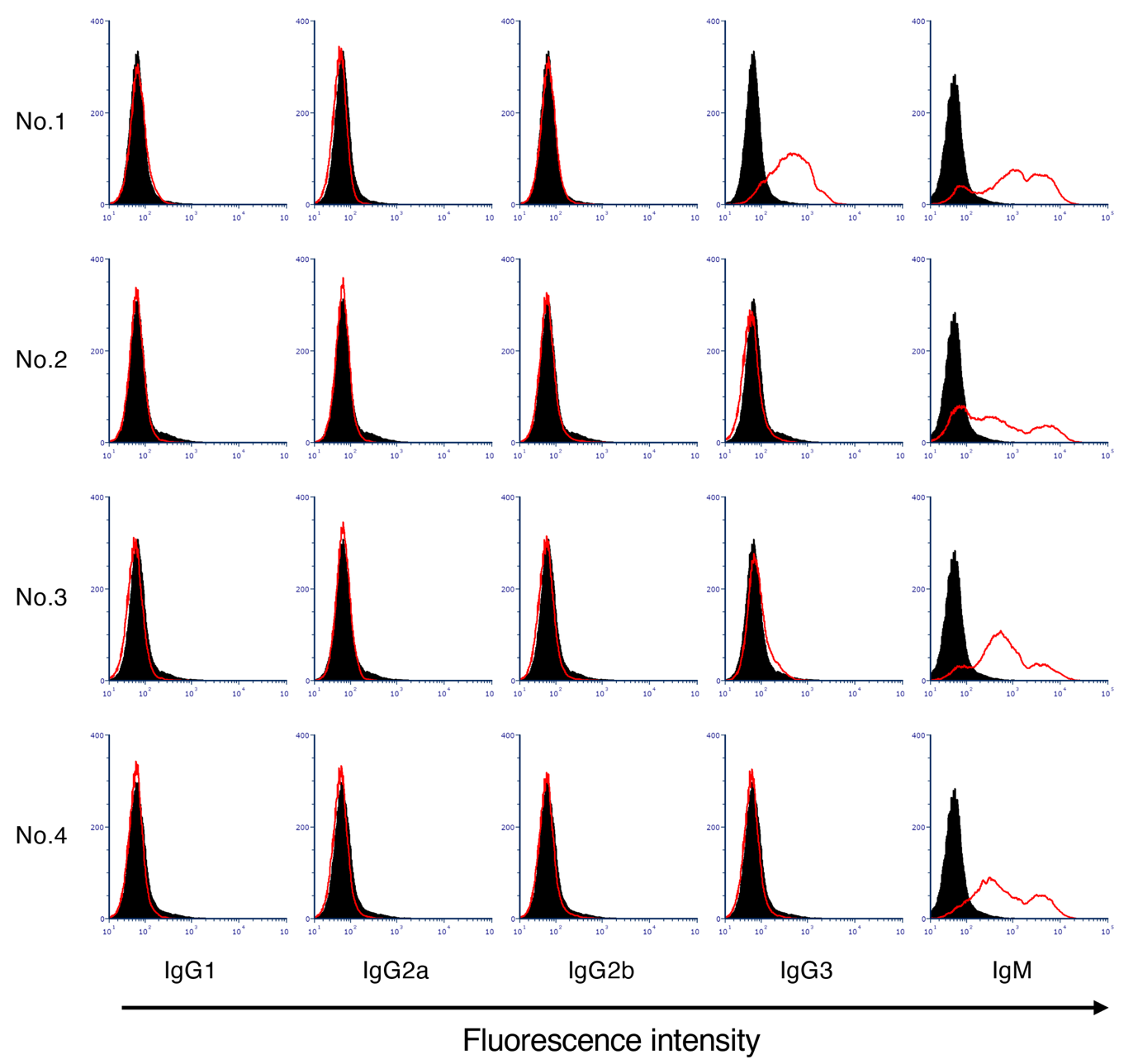


Fig. S4

**FCM analysis images of the reactivity to MCF-7 cells of serum antibodies from mice immunized with Globo-H–containing liposomes.**

Nos. 1, 2, 3, and 4 represent serum samples from different individual mice. The X- and Y-axes indicate the fluorescence intensity and event number, respectively. The fluorescence intensity values of cells reacted with each serum sample are indicated by red lines. Controls for background monitoring were prepared using standard total mouse IgG and IgM and the corresponding fluorescently labeled secondary antibodies (dark shading).


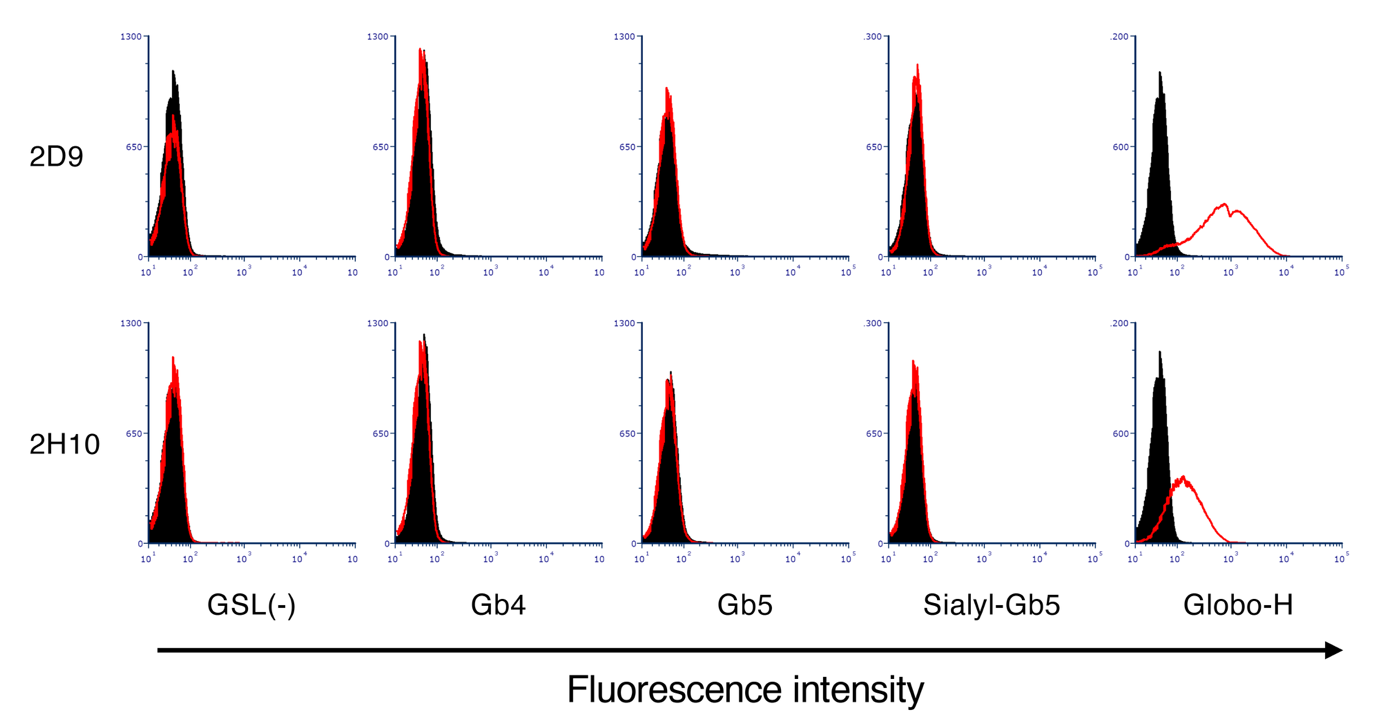


Fig. S5

**FCM analysis images of the reactivity of 2D9 (upper panel) and 2H10 (under panel) mAbs to GSL-containing liposomes.**

Gb4, Gb5, Sialyl-Gb5, and Globo-H indicate liposomes containing the respective GSLs. GSL(−) indicate GSL-free liposomes. The X- and Y-axes indicate the fluorescence intensity and event number, respectively. The fluorescence intensity values of liposomes reacted with each antibody are shown by red lines. Controls for background monitoring were prepared using standard mouse IgG3 (for 2D9) and IgM (for 2H10) and the corresponding fluorescently labeled secondary antibodies (dark shading).
